# Neurorestoration Approach by Biomaterials in Ischemic Stroke

**DOI:** 10.3389/fnins.2020.00431

**Published:** 2020-05-12

**Authors:** Noelia Esteban-Garcia, Cristina Nombela, Javier Garrosa, Fernando J. Rascón-Ramirez, Juan Antonio Barcia, Leyre Sánchez-Sánchez-Rojas

**Affiliations:** ^1^Regenerative Medicine and Advanced Therapies Lab, Instituto de Investigación Sanitaria San Carlos, Clínico San Carlos Hospital, Madrid, Spain; ^2^Department of Biological and Health Psychology, Universidad Autónoma de Madrid, Madrid, Spain; ^3^Neurosurgery Department, Clínico San Carlos Hospital, Madrid, Spain; ^4^Chair of Neurosurgery Department, Clínico San Carlos Hospital, Madrid, Spain

**Keywords:** neurorestoration, repair, biomaterials, stroke, cell therapy

## Abstract

Ischemic stroke (IS) is the leading cause of disability in the western world, assuming a high socio-economic cost. One of the most used strategies in the last decade has been biomaterials, which have been initially used with a structural support function. They have been perfected, different compounds have been combined, and they have been used together with cell therapy or controlled release chemical compounds. This double function has driven them as potential candidates for the chronic treatment of IS. In fact, the most developed are in different phases of clinical trial. In this review, we will show the ischemic scenario and address the most important criteria to achieve a successful neuroreparation from the point of view of biomaterials. The spontaneous processes that are activated and how to enhance them is one of the keys that contribute to the success of the therapeutic approach. In addition, the different routes of administration and how they affect the design of biomaterials are analyzed. Future perspectives show where this broad scientific field is heading, which advances every day with the help of technology and advanced therapies.

## Background

Stroke is one of the most important health problems worldwide. Ischemic stroke (IS) constitutes 85–90% of the casuistry among the types of stroke and is the leading cause of disability in people over 65 years of age worldwide (Ghuman and Modo, 2016). Due to the epidemiological importance and the big socio-economic expenditure involved, it is priority advance in its prevention, control, and treatment (Kalaria et al., 2016; Benjamin et al., 2017). The ischemic injury is caused by an interruption of blood supply in one or more cerebral blood vessels triggering a set of dynamic processes that affect all brain cells and extracellular matrix (ECM) deteriorating the “glioneurovascular niche” (Boisserand et al., 2016).

The pathophysiology of IS lies in the restriction or reduction of the supply of oxygen, glucose, and nutrients in the affected brain area. The ischemic cascade begins while there is arterial obstruction causing accidental cell death of core cells damaging tissue irreversibly. This process is accompanied by events of glutamate excitotoxicity, oxidative stress, and neuroinflammation, which affect the homeostatic functioning of the neurons in the affected tissue. The combination of all of them induces permanent brain lesions (Taylor et al., 2008; Thundyil and Lim, 2015; Thornton et al., 2017). However, there are regions near the nucleus or ischemic penumbra (IP) that have had access to a collateral blood circulation, being able to partially counteract the energy deficit (Fisher and Albers, 2013; Gavaret et al., 2019).

This review will briefly address the limitations and consequences that arise after the stroke, the endogenous repair mechanisms activated by the brain damage itself, how to enhance these mechanisms through tissue engineering and the incorporation of exogenous cells or growth factors.

## Stroke Stage

The pathological picture of IS is aggravated by anatomical and metabolic limitations of the central nervous system (CNS) itself: the glucose and glycogen deposits of the brain are only able to cover the brain’s energy requirements for a brief period and the selective nature of the barrier hematoencephalic (BBB) limits the rate of transfer of molecules from the bloodstream to the brain, restricting access to the necessary substrates for cellular metabolism (Lipton, 1999; Bang et al., 2009).

Therefore, the time factor is decisive to minimize the extent of damaged brain tissue around the core. The period in which it is possible to reduce the impact of IS (therapeutic window) ranges from re-perfusion to 6–24 h, which is very restricted (Crunkhorn, 2018). The positive feedback mechanism of bioenergetic failure, oxidative stress, and inflammatory reaction after IS lead to an adverse microenvironment, incapacitating potentially recoverable cells, to resume their functions. Consequently, it causes damage to the ECM, accumulation of extracellular fluid (Baeten and Akassoglou, 2011), and activation of microglia, macrophages, and astrocytes (Denes et al., 2007; Lalancette-Hebert et al., 2007).

Oligodendrocytes and damaged neurons produce a change in the chemical composition of the extracellular medium that serves as a chemotactic stimulus for microglia and astrocytes. Glial cells alter the pH of the medium and produce an exacerbated inflammatory response by secreting pro-inflammatory cytokines, tumor necrosis factor (TNF-alpha), and interleukin (IL1) (Minami et al., 1992; Lambertsen et al., 2005; Dugue and Barone, 2016). Furthermore, they require a long period to phagocyte and degrade the wastes of dead cells. However, it has been shown that microglial activation can maintain and support neuronal survival by secreting anti-inflammatory and neurotrophic factors (Streit, 2002; Harry et al., 2004). In several studies, it has been shown that microglia promote neurogenesis, guiding neuroblasts to the site of injury (Ziv et al., 2006; Fitch and Silver, 2008; Thored et al., 2009).

In addition to the immune response, astrocytes are activated, modifying their phenotype (reactive astrocytes) to express a series of inhibitory factors, such as cytokines and chemokines, converting the damaged area into a region of restricted transit of molecules and axonal cone growth (Wieloch and Nikolich, 2006; Fitch and Silver, 2008; Paixão and Klein, 2010). Besides, reactive astrocytes begin to synthesize large amounts of chondroitin sulfate proteoglycans, forming a fibrous and acellular membrane, known as a glial scar, which acts as a physical barrier (Busch and Silver, 2007; Yoshioka et al., 2010). This rapid reaction of the microglia and astrocytes has in order to contain the damage and prevent it from spreading, quickly sealing the open path.

## Spontaneus Neuroreparation Process

In the first instance, it is necessary to distinguish between the concepts of repair and regeneration. The first of these refers to the replacement of lost cells in damaged tissue with new cells suitable for the niche; while the second refers to the replacement of injured tissue with homologous tissue, which does not occur in the brain (Modo and Badylak, 2019).

After the pathological events, scientific evidence of the spontaneous activation of endogenous repair processes of the damaged area in the ischemic brain that function as compensatory mechanisms has been described (Arvidsson et al., 2001, 2002; Lindvall and Kokaia, 2015). Among them we can highlight two, the neurogenesis and angiogenesis processes.

### Neurogenesis

Neurogenesis is defined as the process by which new neurons are formed from precursors, located in specific areas known as neurogenic niches, from where they migrate, differentiate, and integrate into their destiny to become functional neurons (Ohab and Carmichael, 2008). Despite that the subventricular zone (SVZ) is not the only neurogenic niche in the adult brain, it is the main source of precursors that reach the ischemic zone. The transient and spontaneous increase of parents is produced by a shortening of the cell cycle, beginning at 2 days and reaching the maximum in 2 weeks after the beginning of the damage returning to its basal levels at 6 weeks after it (Zhang et al., 2001; Thored et al., 2006; Zhao et al., 2008).

It has been described that neuroblasts, which physiologically migrate via the migratory rostral route (MRV) to the olfactory bulb, are redirected to the injured area (Arvidsson et al., 2001; Ming and Song, 2005; Ohab and Carmichael, 2008). Ectopic migration begins 3 or 4 days after damage ischemic and remains up to 4 months after it. The redirection is produced by stimuli sent from the ischemic zone through two routes: through changes in the composition of the cerebrospinal fluid (CSF) or through the diffusion of signals through the blood vessels (Christie and Turnley, 2012; Lindvall and Kokaia, 2015). Factors involved in the redirection of neuroblasts, such as brain-derived neurotrophic factor (BDNF), stromal cell-derived factor-1 (SDF-1α) and its CSCR4 receptor, monocyte chemoattractant protein-1 (MCP-1), and metalloprotease (MMP-9) matrix released by neuroblasts themselves (Thored et al., 2006; Bagley and Belluscio, 2010).

### Angiogenesis

During the IS, some brain areas are supported by access to collateral flow from pre-existing anastomosis. After ischemic damage, the reduction in blood flow leads to both acute and chronic vascular remodeling. This vascular repair process adds to that of neurogenesis to promote the recovery of damaged tissue (Thored et al., 2006).

In recent years, the data obtained from magnetic resonances in experimental models of ischemic damage have revealed vascular remodeling processes in both acute and chronic phases. This process has been observed due to an increase in cerebral blood volume (CBV) in its late phase induced by spontaneous stimulation of angiogenesis (Arai et al., 2009; Carmeliet and Jain, 2011; Liu et al., 2014). Cerebral vasculature has been defined as a key factor in the progress of pathological processes and in homeostasis. The bidirectional connection between the nervous system (NS) and the vascular system is strongly established in the CNS (Xu et al., 2017). On the one hand, the NS depends on the integration, communication, and functionality of the different vascular cellular phenotypes for their metabolic and nutritional support; and in turn, the vascular system requires nerve innervation for different regulatory mechanisms, such as vasodilation and vasoconstriction (Uhrin, 2019).

Cerebral angiogenesis is closely regulated by mediating angiogenic factors and the local microenvironment. Recently, these factors have been shown to play an essential role in endothelial cell migration, cell identity, and growth and the regulation of BBB; being involved, in addition, in the alignment of vessels-nerves and nerves-artery in the brain (Arai et al., 2009; Carmeliet and Jain, 2011; Liu et al., 2014). Among these angiogenic factors, the following stand out: (i) the endothelial growth factor (VEGF) that stimulates angiogenesis through VEGF-2 receptors; (ii) the netrins that act as bifunctional signals of attractant or repellent guidance depending on the receptors expressed by the different cell types; (iii) fibroblast growth factor (FGF) that maintains vascular integrity; and (iv) platelet-derived growth factor (PDGF) that is crucial for the maturation and functioning of blood vessels (Carmeliet and Jain, 2011).

Therefore, the angiogenesis process has been postulated as a key restorative mechanism in the response to an ischemic event that participates in functional recovery.

Despite the spontaneous stimulation of neurogenesis and angiogenesis triggered by the body itself to restore the damaged area, there are very few precursors that manage to reach the target; and even less, to mature and repopulate the area (Arvidsson et al., 2002). This failure may be due to the inflammatory environment (Kahle and Bix, 2013), to the deficit of functional connections, and the necessary trophic support (Ming and Song, 2015). Therefore, the recovery of neural function depends, for the most part, of the ability of nearby unaffected neurons to generate new synapses, which is known as neuronal plasticity (Wieloch and Nikolich, 2006; Paixão and Klein, 2010).

## Powering Neurorepair Process

The chronification of gliosis and inflammation in the twilight zone makes endogenous repair strategies difficult (Wieloch and Nikolich, 2006). This is the perfect time to establish strategies that enhance and complement endogenous repair mechanisms in order to partially rebuild the tissue damaged and restore neurological function (Arai et al., 2009; Fisher and Albers, 2013; Liu et al., 2014; Thundyil and Lim, 2015). The development of tissue engineering in the brain with ischemic injury has positioned itself as a great promise to overcome these limitations and replace tissue loss (Modo and Badylak, 2019).

### Why Use Biomaterials?

Ischemic brain injury causes a reduction in brain volume (atrophy) that includes the elimination of ECM (Moreau et al., 2012). This is a current challenge for the effective treatment of stroke. Therefore, a support structure such as bioscaffolds is required. Biomaterials are natural or synthetic 3D polymer networks (natural or synthetic ED polymer networks) that provide a suitable environment for cells to survive, proliferate, and differentiate, facilitating the formation of ECM (Ghuman et al., 2016) and for cells to be able to restore their function. These two facts are keys to neurorestoration.

The first biomaterial utility is to offer structural support in an injury that leads to loss of parenchyma, thus facilitating the invasion of the different support molecules and the new endogenous cells. This support allows these to overcome the glial scar generated and penetrate the lesion (Meng et al., 2014; Modo et al., 2018).

As for its second utility, in addition to supporting the physical migration of cells, it is also necessary that inductive signals from the biomaterial be produced to initiate migration and cell invasion. Therefore, biomaterials are being widely used as controlled releases of drugs, cells, and exogenous molecules. The advantage of this fact is that they are carriers of the bioactive molecules up to the therapeutic target, being able to control the rate of release (Massensini et al., 2015).

In addition, biomaterials can act as a protective barrier for these molecules against the adverse microenvironment that exists in ischemic tissue. This protection supposes an increase of the effectiveness of the treatment in the target, although it is not eternal, since when the biomaterial degrades, its protection ceases.

### Criteria to Take Into Account to Define Your Design

It is important to consider the chemical and mechanical properties that the biomaterial presents, since the success of its functionality and the fate of the transplanted bioactive molecules will depend on them.

#### Biocompatibility

The first issue to highlight is that it is biologically accepted by the host tissue, producing a minimal immune and inflammatory response and that, in addition, it is able to maintain its benefits during its useful life (Mitragotri and Lahann, 2009; Wang, 2013). The long-term biocompatibility of the material with the Host tissue marks the effectiveness of implantation. The degree of astrocyte and microglial reaction that may appear around the biomaterial is used in *in vivo* studies to terminate the degree of biocompatibility (Fournier et al., 2003).

#### Biodegradation

The degradation rate of biomaterials is one of its most important chemical properties, since it allows the release of the bioactive molecules it contains and the structural remodeling of the neural network. There are different formats of presentation of the biomaterial according to the polymerization process used; for example, hydrogels are usually designed for slow degradation, helping or favoring exogenous cells to develop their own ECM (Mano et al., 2007). However, the higher their biodegradation rate, the more likely it is that a rejection reaction will occur. Therefore, it is convenient to find a balance between degradation rate and functionality (Perez-Garnes, 2015).

#### Functionality

The functionality of the scaffold is defined by its composition, the place of implantation, the route of administration, the fate of the exogenous cells that house and/or the release of the drug, which is achieved through its chemical and mechanical properties.

### Composition

#### Synthetic

One of the most outstanding advantages of synthetic biomaterials is the possibility of obtaining a homogeneous batch production, that is to say, precisely elaborating certain physical-chemical properties (Busscher et al., 2012; Rimondini et al., 2015; Ghuman and Modo, 2016). Uniform manufacturing translates into greater control of their degradation rate, being optimal candidates to be carriers of drugs or small molecules with controlled release after administration. Ultimately, this advantage results in the reduction of the variability in the immune response generated in the host.

Synthetic biomaterials have been widely used for other pathologies but taking into account the characteristics of the brain as host tissue, and its slow rate of degradation; have not been the best candidates to treat the stroke. However, the most widely used synthetic compounds have been polymers of polylactide (PL), polyglycol (PG), polycaprolactone (PCL), and co-polymers of lactide and glycolide (PLGA). This last compound has been used in nanoparticles form, which has positioned it as one of the best synthetic biomaterials to carry substances even in the brain. A recent paper is the one published by Jeong et al. (2019), who encapsulated erythropoietin in PLGA nanoparticles and cholic acid, because it crosses the blood–brain barrier among other advantages.

The formation of bioscaffold can be carried out, by loading the molecules of interest at the site of the lesion itself or by previously cross-linking with the material itself (Yang et al., 2006; Wong et al., 2007; Dash and Konkimalla, 2012).

Another synthetic polymer commonly used is polyethylene glycol (PEG), resistant to protein degradation. A recent study has been published using PEG conjugated urokinase nanogels (PEG-UK) demonstrating that administration of PEG-UK outside the usual therapeutic window could still exert protective effects in permanent middle cerebral artery occlusion (pMCAO) rats through maintenance of integrity of BBB and the inhibition of apoptosis and excito-neurotoxicity (Cui et al., 2020). Authors, as Balasubramanian et al. (2020), have recently published a study based on silicone nanoparticles, with the aim of promoting the migration of endogenous neuroblasts in post-stroke. This type of component has been less used, but it is not less valid and beneficial.

Other studies inspired by natural platelets (PLTs) and their role in targeting adhesion to the damaged blood vessel during thrombus formation have fabricated a biomimetic nanocarrier comprising a PLT membrane envelope loaded with l-arginine and γ-Fe2O3 magnetic nanoparticles (PAMNs) for thrombus-targeted delivery of l-arginine and *in situ* generation of nitric oxide (NO); for the early treatment of IS (Li et al., 2020).

Because the cells are not able to adhere directly to it, recent studies have used combinations of natural compounds such as hyaluronic acid (HA) or gelatin, thus optimizing their characteristics (Sharma et al., 2015; D’souza and Shegokar, 2016). However, synthetic biomaterials have a limited capacity to induce endogenous repair responses, so their majority use has been for prostheses and implants (Koupaei et al., 2015; Yuan et al., 2019).

#### Natural

Unlike synthetic biomaterials, natural biomaterials are compounds present in the ECM, which increases biocompatibility with the host tissue and the restoration of the adverse microenvironment. The ECM of the nervous tissue constitutes 20% of the cerebral parenchyma and, its functions are directly related to the maintenance of the structure and the cellular signaling (Stabenfeldt et al., 2006; Reing et al., 2009). The objective of the natural scaffolds is to implant in the damaged tissue an ECM “transient or permanent substitute” that facilitates cell growth to form, again, the three-dimensional structure of the tissue to be repaired (Crapo et al., 2012).

The most widespread natural compounds for application in the restoration of tissue defects and improvements in the adverse microenvironment are fibrin, HA-methylcellulose, chitosan, and collagen (Hopkins et al., 2013; Medelin et al., 2018; Osama et al., 2018).

The combination of hyaluronic acid + methyl cellulose (HAMC) has been used for the first time by Gupta et al. (2006) and has been widely used in models of stroke, spinal cord injury, and retinal degeneration (Ho et al., 2019). One of the last published articles has been a study developed by Tuladhar et al. (2020), where they have used this HAMC combination as a vehicle to release cyclosporine and erythropoietin, to promote functional recovery in stroke.

In most cases, they have been used in combination with exogenous cells that enhance endogenous repair mechanisms (Moshayedi and Carmichael, 2013). One of the latest articles published by Fernandez-Serra et al. (2020) is based on a fibroin biomaterial with the same objective, that of recovering post-stroke function, this time encapsulating mesenchymal stem cells. Other compounds, such as alginate, have been used in microspheres (Cui et al., 2013) or recently, to encapsulate cells in combination with synthetic compounds (Islam et al., 2018).

Two of the most abundant compounds in ECM are collagen and HA, which is why their use in biomedicine has been extended in the last decade. Collagen has mechanical resistance and immunogeneity, and fragments derived from active collagen contribute to biological activities such as growth, differentiation, and cell migration, which has facilitated its use in various studies with rodent models in the form of hydrogel (Cross et al., 2010). In the study conducted by Yu et al. (2010) demonstrated in an ischemic mouse model, an increase in cell survival, synapse formation, and an improvement in neural function by implanting a collagen hydrogel combined with neural stem cells (NSCs).

The other natural compound, with similar characteristics, found in the ECM of the CNS is HA. The enzymes responsible for their formation, and therefore, their size and molecular weight are hyaluronan synthases (HAS) (Brecht et al., 1986; Weigel et al., 1997), these are found in the cell membrane of fibroblasts, keratinocytes, chondrocytes, and specialized connective tissue cells. The molecular weight of HA is directly related to its biological functions; specifically, HA of ≥60 kDa is attributed with non-stick properties for cells (Brecht et al., 1986). These allow astrocytes to be kept in a non-reactive state and inhibit the formation of glial scar (Lin et al., 2009; Khaing et al., 2011). In addition, their anti-inflammatory properties and their support for cell survival have been demonstrated (Jiang et al., 2014). In an *in vivo* study of mouse model with occlusion of the middle cerebral artery occlusion (MCAO), in which an HA hydrogel with bioactive molecules (VEGF or angiopoietin-1) was implanted, observed good biocompatibility with brain tissue and increased angiogenesis around the implanted hydrogel (Ju et al., 2014). In this line, other authors have combined an HA hydrogel with exogenous cells, neural progenitor cell (NPCs) (Zhong et al., 2010), or adipose-derived stem cells (ASCs) (Sanchez-Rojas et al., 2019), observing a lower infiltration of reactive cells in the biomaterial and an increase in neural precursors in the area of the lesion.

In Table 1 shows a scheme with the main synthetic, natural, and mixed biomaterial compositions have been described in this review.

**TABLE 1:** Summary of natural and synthetic components of biomaterial.

**References**	**Biomaterial**	**Composition**	**Experimental model**	**Main findings**
Meng et al., 2014	Synthetic	Synthetic fibronectin peptide (PRARIY)	Middle cerebral artery occlusion (MCAo) in Sprague–Dawley (SD) rats	- Reduction of infarction size - Significantly functional outcome - Decrease in apoptosis
Modo et al., 2018	Synthetic	Human neural stem cells (HNSCs) on vascular endotelial growth factor (VEGF)- releasing PLGA microparticles	MCAo in SD rats	- Attraction of endothelial cells from the host, establishing a neovasculature interspersed with NSCs
Wong et al., 2007	Synthetic	Poly (ε-Caprolactone) and PLGA polymer	Acute traumatic brain injury (TBI) in SD rats	- Decrease in astrocytic activation - Promotes neural ingrowth - Prevention of the enlargement of the defect
Medelin et al., 2018	Synthetic	Chitlac (A derivative of chitosan)	Primary culture of hippocampal neurons of postnatal (P2–P3) SD rats	- Induces growth and synapse formation *in vitro*
Ju et al., 2014	Synthetic	Hyaluronic acid (HA) hydrogel + PLGA microspheres containing VEGF and Angiopoietin-1 (Ang-1)	MCAo in C57BL/6J mice	- High rate in angiogenesis - Behavioral improvement - Formation a suitable niche for neural restoration
Jeong et al., 2019	Synthetic	Cholic acid-coated poly lactic-co-glycolic acid (PLGA) nanoparticles loaded with EPO (EPO-CA-NPs)	Middle carotid artery occlusion and reperfusion (MCAO/R) technique in rats	-Able to cross the BBB - Reduction in the extent of the infarct volume and cellular apoptosis - Better performance on sensorimotor phenotype than EPO alone
Cui et al., 2020	Synthetic	Polyethylene glycol conjugated urokinase nanogels (PEG-UK)	Permanent MCAO (pMCAO) in adult male SD rats	- Amelioration of the severity of neurological deficits - Decrease in the infiltration of inflammatory cells and the concentration of interleukin 1β (IL-1β) and tumor necrosis factor-α (TNF-α) in the brain parenchyma - Inhibition of apoptosis and excito-neurotoxicity
Li et al., 2020	Synthetic	Natural platelet (PLT) membrane envelope loaded with l-arginine and γ-Fe_2_O_3_ magnetic nanoparticles (PAMNs)	Photochemical cortical ischemic stroke in C57BL/6 mice	- Rapid targeting to ischemic stroke lesions - Promotes vasodilation to disrupt the PLT aggregation - Recovery of blood flow
Ghuman et al., 2016	Natural	Extracellular matrix (ECM) purified from porcine urinary bladder (collagen, fibronectin, decorin, laminin)	MCAo in SD rats	- Promotes host cell infiltration - Retention of the hydrogel within the cavity of the lesion - Antiinflammatory properties
Meng et al., 2014	Natural	High-molecular weight HA (HMW-HA) hydrogel	4 × 2 × 2 mm^3^ cortical lesión created in SD rats	- Reduction in glial scar thickness - Decrease in astrogliosis marker GFAP
Meng et al., 2014	Natural	Laminin-incorporated HA (LN-HA) hydrogel	Cortical defects induced mechanically in SD rats	- Support cell infiltration and angiogenesis - Inhibit the formation of the glial scar - Promotes neurite regrowth
Crapo et al., 2012	Natural	ECM purified from porcine tissues (collagen, fibronectin, decorin, laminin)	PC12 cell line	- Stimulation of cell proliferation
Osama et al., 2018	Natural	Silk hydrogel (4% w/v) + mesenchymal stem cells (MSCs)	MCAo in SD rats	- Good space conformity in the ischemia cavity
Moshayedi and Carmichael, 2013	Natural	Hyaluronan-heparin-collagen hydrogel + neural progenitor cells (NPCs)	Photothrombotic ischemia in C57BL/6 mice	- Improvement of NPCs survival into the infarct cavity after stroke
Islam et al., 2018	Natural	Alginate-collagen microspheres containing fibroblast growth factor 2 (FGF-2)	Zebrafish embryos	- Increase in therapeutic angiogenesis
Yu et al., 2010	Natural	Collagen type I + neural stem cells (NSCs)	MCAo in Wistar rats	- Survival of the NSCs engrafts - Synapsis formation - Differentiation of NSCs
Zhong et al., 2010	Natural	Hyaluronan-heparin-collagen hydrogel + NPCs	Cortical photothrombotic stroke in C57BL/6J mice	- Improvement in NPCs survival *in vitro* and into the infarct cavity (*in vivo*)
Sanchez-Rojas et al., 2019	Natural	HA + adipose stem cells (ASCs)	Middle cerebral artery thrombosis with FeCl_3_ in athymic mice	- Increase in cell proliferation and neurogenesis at subventricular zone (SVZ) - Angiogenesis and less inflammatory reaction to the graft
Tuladhar et al., 2020	Natural	Hydrogel drug depot, comprised of hyaluronan and methylcellulose (HAMC) containing cyclosporine and erythropoietin (CsA + EPO)	Endothelin-1 stroke in male SD rats and male Long-Evans rats	- Long term stability in the brain - Cyclosporine increased plasticity in the striatum while erythropoietin stimulated endogenous NSPCs
Fernandez-Serra et al., 2020	Natural	Silk fibroin hydrogels-encapsulated MSCs	MCAo in adult male CD-1 mice	- Promotes the survival of intracerebrally implanted MSCs - Improvement of functional outcomes over time in the model of cortical stroke

## Choosing Administration Route

The anatomical limitations of the CNS, such as the skull and the BHE, restrict the passage of molecules and their accessibility. It is necessary to take these particularities into account to determine which route of administration is ideal for each treatment (Bible et al., 2009a). In addition, the chosen route will define the biomaterial format. For example, some brain areas of interest can be found at great depth, covered with functional tissue that should not be altered. In these situations, it is convenient to use the intracerebral route to guarantee the correct implantation in the therapeutic target (Bible et al., 2009b; Ullah et al., 2017). For this route, it is necessary to use cannulas, in the cases of solid biomaterials or Hamilton syringes for the administration of nanoparticles (Ullah et al., 2017), microspheres (Bible et al., 2012), liquid neurospheres or hydrogels, which are administered before polymerization. This last pharmaceutical form has acquired great interest in *in vivo* studies, due to its easy and minimally invasive administration. It is inoculated by a Hamilton and polymerized, approximately, 8 min later; offering a structural 3D network for endogenous cells (Tate et al., 2001).

In the case that the therapeutic target is in the orbitofrontal cortex, the intranasal route is the one that offers the most advantages, due to the excellent conditions of the nasal mucosa for its absorption and the direct connection with the ethmoid bone. Currently, there are more and more studies in which biomaterials are used in neurospheres through this route, due to their effectiveness, safety, and speed (Yongjun et al., 2011; Wei et al., 2013; Yan-hua et al., 2015).

Also, it is possible to administer these low molecular weight nanocomposites through the intravenous route; however, it has been shown that the dose that reaches the cerebral target is insufficient, and therefore, it is necessary to increase the dose and find adverse effects (Tosi et al., 2019).

## Enhancing Cellular Attraction by Cell Therapy and Trophic Factors

At present, many studies use the combination of cell therapy or bioactive molecules and biomaterial to improve its invasion and colonization in the host tissue. In addition, the use of these types of exogenous cells or bioactive molecules has been shown to have an effect *per se* on damaged tissue (Lam et al., 2014; Sanchez-Rojas et al., 2019).

Despite the inflammatory reaction produced in the tissue that inevitably occurs when implanted, it has been shown that the effect of transplantation stimulates endogenous neural precursors through chemoattractant signals, promotes neuroprotection, and modulates neuroinflammation (Orive et al., 2009; Dibajnia and Morshead, 2013). As has been shown, stem cells do not integrate into the tissue, so their use is restricted to their trophic potential for 2 or 3 weeks (Modo and Badylak, 2019). Other authors opt for the encapsulation of trophic factors directly, such as VEGF or BDNF (Bible et al., 2012; Guan et al., 2012; George et al., 2018). Both strategies have the common objective of promoting neuroreparation processes (Erba et al., 2010).

After an ischemic event, the glial scar formed isolates the lesion from the rest of the parenchyma. In the study conducted by Zhou et al. (2015) showed that the administration of ASCs significantly suppressed the expression of the ionized calcium binding adaptor molecule 1 (Iba1) marker and glial fibrillary acidic protein (GFAP) marker compared to the control group. On the other hand, focal cavitation produced after the ischemic event is also a handicap for the effective treatment of stroke. Therefore, the use of biomaterials combined with cell therapy facilitates the establishment of a line of communication between the healthy parenchyma-biomaterial-lesion and favors the microenvironment (Martínez-Ramos et al., 2012; Pérez-Garnes et al., 2014).

As already mentioned, the chemical properties of the biomaterial affect its mechanical properties and therefore, the bioactive molecules or exogenous cells that are inside. The stiffness of biomaterials affects cell proliferation and differentiation *in vivo* (De Santis et al., 2011). Biomaterials with intermediate stiffness have been shown to improve cell proliferation (Leipzing and Shoichet, 2009). In this line, mesenchymal stem cells respond differently in gels of different viscosity (Engler et al., 2006). Therefore, it is important to choose the biomaterial format depending on what you want to combine with.

Other considerations to take into account for clinical translation are safety and efficacy. The implementation in the SNC entails safety specifications so that the least number of adverse effects occur. Delivery directed to the target to avoid tissue displacement or the generation of cavitation is of paramount importance. On the other hand, when performing the intracranial implant, it is necessary to control the speed and intracranial pressure since they could cause bleeding and an exacerbated inflammatory response. In addition, the use of exogenous cells or bioactive molecules produces a proliferative response, so it is necessary to control that no neoplastic growths occur (Eckert et al., 2013; Xu et al., 2017).

Table 2 shows a brief summary with the advantages and disadvantages of the different strategies that have been described in this review.

**TABLE 2:** Summary of advantages and disadvantages of the proposed strategies.

**Strategy**	**Advantages**	**Disadvantages**
Synthetic biomaterial (PL), (PG), (PCL), (PGLA)	Degrading rate control Homogeneous production Reducing variability in immune response	Limited ability to induce endogenous repair responses
Synthetic biomaterial combined with natural compounds	Resistance to protein degradation Optimizing repair features	Hydrophobicity superficial
Collagen biomaterial	Contributing to growth, differentiation, and cell migration	Immunogeneity Low mechanical resistance
Alginate biomaterial	Biodegradable Hypoallergenic	Combined with synthetic compounds for greater consistency
Hyaluronic acid biomaterial	Anti-inflammatory properties Support for cell survival biocompatibility Non-stick properties	Easily degradable Possible formation of fibrosis
Biomaterial combined with exogenous cells	Less infiltration of reactive cells into the biomaterial Increase in neural precursors, modulates neuroinflammation, promotes neuroprotection	Possible neoplastic formation Cells do not integrate into the tissue
Biomaterial combined with bioactive molecules	Promoting neuroreparation processes Improves invasion and colonization of host tissue	Its use is restricted to 2–3 weeks

## Future Perspectives/Next Steps

Scientific advances place exosomes or extracellular vesicles as the new candidates to be used to improve their colonization and integration into tissue. These molecules are much smaller than a cell and have a key role in intercellular communication. These characteristics are sufficient to develop biomaterials in which to encapsulate them, with the advantage not only of being able to encapsulate a high number of exosomes in each microsphere, but to avoid the adverse reactions associated with the stem cells since, at least so far, has been described to have tumorigenic capacity (Chen and Chopp, 2018).

In addition, research is continuing how to deliver neurospheres or nanoparticles to deep and distal areas of the cerebral parenchyma from the intranasal route. Apparently, it is a route of minimally invasive administration that allows direct access to the brain, avoiding the anatomical limitations of the CNS. However, so far, no remains of these biomaterials have been found farther from the orbitofrontal cortex. Perhaps there is an intracerebral circulation that we still do not know today (Shah et al., 2015).

One of the great challenges is in bioprinting. Currently, very advanced 3D printer technology is being developed that has great advantages, such as homogenizing lots of biomaterials. It is possible to manufacture or print many biomaterials of small dimensions with precision and, in addition, all are exactly the same. It is also possible to make homogeneous mixtures of a drug in the biomaterial thread, instead of encapsulating it. The advantage of this method is the control over the mixture, being possible different concentrations of drug in the same biomaterial, or even of several compounds that are degraded simultaneously or staggered. The pharmaceutical forms are being reinvented. On cell therapy, printers have been developed that directly print the cells of interest in a certain position. This technology requires a high degree of sterility and its price is still very expensive. However, there is no doubt that the future is in these techniques (Norotte et al., 2009; Hsieh and Hsu, 2015).

Personalized medicine will be imposed in the future given the variability of brain damage and diseases; and biomaterials can adapt to this new approach. An exclusive design for a specific lesion is possible, with a volume of affection, a location, and very specific particularities.

What is already a certainty today is the safety of many of the components of biomaterials and cells (for example, adipose cells) supported by biomaterials in animals (Zhao et al., 2019; Kupikowska-Stobba and Lewińska, 2020; Otake et al., 2020). These results led us to argue that the gap between animals and humans in this context will be closed soon. Indeed, recent clinical trials have been conducted in Phase IIb to support the use of restorative cells plus natural biomaterials (alginate encapsulates) in neurodegenerative conditions (e.g., Parkinson’s disease) that achieve promising results (Snow et al., 2019).

## Conclusion

Despite the advances in the design, development and manufacture of biomaterials to favor neural restoration and the microenvironment, it is still a great challenge today. Minimally invasive techniques are sought to release cells, trophic factors, or drugs that potentiate spontaneous neuro-restaurant mechanisms. At the same time, it is sought that the biomaterial arrives and/or remains on the therapeutic target; and to be kept there during its degradation causing the least possible inflammatory reaction. Also, that it is colonized by endogenous cells, facilitating access to support cells to the center of the lesion and crossing the glial scar.

Combinations of biomaterials made of natural and synthetic compounds offer the advantages that both provide. And with the development of new forms such as microspheres, nanoparticles, liquid hydrogels that polymerize within a few seconds, or solids offer many possibilities for personalized treatments.

The objective and function to be achieved, the route of administration, and the limitations that exist to design a successful biomaterial must be defined.

## Author Contributions

All authors substantially contributed to the manuscript design, they critically reviewed it and gave their final approval. NE-G, CN, and JG were in charge of the bibliographic search and writing. NE-G and LS-S-R conceived the structure and specified the content of the article. JB and FR-R contributed with their medical perspective and knowledge, specially regarding the administration route and clinical considerations. LS-S-R addressed the “Future Perspectives/Next Steps” and “Conclusion” sections.

## Conflict of Interest

The authors declare that the research was conducted in the absence of any commercial or financial relationships that could be construed as a potential conflict of interest.
